# The Community Health Worker academy: a novel healthcare and public health workforce development model

**DOI:** 10.3389/frhs.2025.1535804

**Published:** 2025-06-26

**Authors:** Nate McCaughtry, Cheryl L. Somers

**Affiliations:** Division of Kinesiology, Health, and Sport Studies, College of Education, Wayne State University, Detroit, MI, United States

**Keywords:** Community Health Worker, CHW, workforce development, training, public health, community health, social determinants of health, health education

## Abstract

As the demand for an expanded Community Health Worker (CHW) workforce accelerates nationwide, the need for training, continuing education, professional development, and experiential field placements (internships and apprenticeships) with quality supervision has expanded dramatically. For example, the US state of Michigan recently received federal approval for the health education and social determinants of health services provided by CHWs to be eligible for Medicaid insurance reimbursement (governmental subsidized healthcare insurance), which has spurred tremendous demand for the CHW workforce by all types of employers. This paper describes a series of events and opportunities that led to the development of the Wayne State University Community Health Worker Academy (CHWA) in Detroit, Michigan, USA. Preliminary evidence suggests that the Academy's model and continuous process improvement strategies are successful. Implications of this model for other organizations involved in expanding and strengthening the CHW workforce development nationally and internationally are discussed, as are implications for career pathway opportunities for CHWs beyond their initial certification**.**

## Introduction

The history of the CHW field, empirical evidence supporting their effectiveness as an integral component of the healthcare and public health workforce, and examples of training models that are common in CHW professional preparation are described below as foundational information that informs and justifies a focus on CHW workforce expansion. Next, the founding and development of the Wayne State University Community Health Worker Academy and the impactful ways in which it serves the unique health and social needs of residents throughout Michigan and particularly in the Detroit metropolitan region is described, as are implications for the future of CHW workforce development and CHW career advancement.

### Evolution of the Community Health Worker field

The Community Health Worker field was born out of a growing recognition that an alternative organization and delivery of healthcare and public health might better respond to the needs, values, and preferences of citizens who have difficulty accessing trusted health education resources and acquiring social services to mitigate factors that influence their social determinants of health, in ways that the traditional medical model of healthcare cannot. While the literature on the history of CHWs is varied, several key United States and international studies have described a consistent historical scope of services and mission of CHWs [e.g., (1–5)]. Across studies and cultural contexts, there is an overlapping theme that CHWs are a unique type of healthcare and public health professional with the knowledge and skills needed to provide effective community outreach and support to advance health equity, capitalize on their status as trusted members of their communities, support residents’ connections to and retention in care and support services, and implement novel strategies to aid communities in accessing health care and other social services that improve social determinants of health, and ultimately reduce chronic disease and other negative health outcomes. Through this lens, it is commonly understood that the CHW workforce has grown substantially, and evidence has increasingly shown the impact of CHWs on individuals’ lives especially those living in underserved communities, primarily through their work in providing community-based, on-going, frequent, and tailored services for residents, especially those most in need of support.

A variety of informal job titles have been used to refer to CHWs [(e.g., Patient Service Representatives, Community Navigators, Health Care Workers, etc.; e.g., (6)]. The commonality is that they all acquire a broad array of knowledge and skills guided by the core consensus national competencies and standards in the USA (https://www.c3project.org/). Regardless of the actual job title, a central feature of CHW work is that they are typically members of the communities in which they serve or they have lived experiences and intimate familiarity with those communities and thus are afforded a much greater level of trust by residents [e.g., (6, 7)]. Specifically, residents are more likely to trust the health education resources and social services that CHWs provide to improve their health outcomes through ongoing education and relationship building, health advocacy, and empowerment. In this way, CHWs are believed to be best positioned to address residents’ complex needs because they have an insider understanding of community dynamics and culture. They understand the common health care and public health access and utilization barriers faced by community members, and their culturally relevant approaches and communications are far more likely to be embraced by residents on such topics as health literacy and chronic disease prevention, detection, and treatment [e.g., (8)].

It is also commonly understood that CHWs have historically been employed in community organizations or health care systems and focus on public health-oriented roles and responsibilities (e.g., as Community Connectors, Health Educators, Linkage to Care experts, etc.). Increasingly, the emerging trend is for CHWs to be involved in integrated care teams at primary care practices, health care systems, public health agencies, mobile health units, accountable care organizations, behavioral health clinics, and other health care and public health organizations alongside other health professionals, together contributing to patients’ healthcare, health literacy, and SDOH (Social Determinants of Health) challenges [e.g., (9)]. Inclusion in multi-disciplinary teams is vital not only to achieve optimal population health impact but also when states mobilize to seek federal authorization to have CHW services reimbursable through Medicaid (e.g., Michigan's recent House Bill 5784, Section 1616). Achieving Medicaid reimbursement not only excites more individuals to become CHWs, but substantially more employers begin seeking to employ CHWs or substantially expand their existing CHW and related staff, thereby ultimately resulting in a growing need for a rapidly expanding workforce. As care team members, CHWs are united with their clinical and traditional healthcare colleagues (e.g., nurses, social workers, physicians, pharmacists, etc.) to execute patient risk assessments, monitor and promote medication adherence, and coordinate community outreach, health information dissemination, provision of social service referrals, and community health advocacy. In many respects, they are dedicated to engaging with community members in ways that have not consistently been done successfully within the context of the traditional medical model of health care practice.

### Empirical support for the CHW profession

The empirical evidence of CHW efficacy and effectiveness is expanding rapidly. Both quantitative and qualitative research have provided evidence that CHW services add a great deal of value to advancing the health of communities, alongside more conventional clinical approaches to healthcare. There is currently worldwide evidence documenting CHWs as critical supports for communities and facilitators of positive community health outcomes (10) and CHW utilization has been particularly well-documented in developing countries [e.g., (11–15)]. A meta-analysis of 30 U.S. studies (16) concluded that CHW involvement in patient medical care intersects “quadruple aims” through community-based (1) clinical services, (2) social service resource connections, and (3) health education and (4) health coaching. These findings have fueled a steadily increasing understanding of the utility and value of CHWs as integral personnel on inter-disciplinary care teams, which has therefore resulted in the need for more widespread and tailored training and continuing education for CHWs in order to expand and strengthen their workforce [e.g., (17)]. These recommendations have been made for several decades, emphasizing the critical nature of the CHW role to change healthcare and public health from “sickness care” into new systems that contribute to better and more sustainable care and outcomes for individuals, families, and communities [e.g., (18)].

Despite these advancements, the expansion of the CHW workforce for many years has occurred slower than might have been expected. The improved patient outcomes have been attributed to CHW services, such as early childhood vaccination rates [e.g., (19)], reduced re-hospitalizations and emergency department visits in low socio-economic status, urban patients with heart failure (20), and mental health symptom reduction [see systematic review by (21)]. The slow proliferation of the CHW workforce is even more curious given the research that has shown CHWs’ contributions to various positive health interventions and patient outcomes, such as increasing access to preventive care, improving patients’ perceptions of their care experiences, reduced healthcare costs, and improved management of chronic disease (22). Additional empirical research has found that CHW services that are integrated into public health and healthcare settings improve cardiovascular disease, hypertension, and other chronic health conditions (23).

According to Knowles et al. (22), dozens of CHW interventions in the U.S. have illustrated the value of integrating CHWs into public health interventions through randomized controlled trials (RCTs) and robust quasi-experimental research techniques such as propensity score–matched cohort studies. Moreover, a growing number of evaluations of CHW interventions that address upstream causes of poor health across varying populations have demonstrated that CHW interventions can lead to improvements across the “triple aim” of health care—improved population health, improved patient care experiences, and reduced healthcare costs—all of which hold promise for advancing health equity (22).

## Background and context

### Common training models and methods

Although in the U.S. there are well-codified national competencies and standards for CHWs, CHW certification training models vary from state to state, based partly on the degree to which various states regulate the CHW workforce and whether insurance reimbursement mechanisms are involved. In fact, CHW certification training programs can also differ a great deal within a single U.S. state. In most states that regulate CHW certifications and/or have secured federal approval to have CHW services reimbursable through Medicaid, state health departments often provide a great deal of flexibility regarding the format and progression of CHW certification programs, provided they adequately teach and assess the national C3 CHW competencies and standards and the duration of certification training programs meet mandated thresholds. For example, the state of Michigan provides some clear certification training mandates, but it also offers organizations a fair amount of autonomy in areas such as instructional design, learning assessments, content progression, and other training components.

In fact, there are several organizations that have developed CHW certification programs according to the requirements established by the Michigan Department of Health and Human Services, for instance the Michigan Community Health Worker Alliance (MiCHWA), Everyday Life Consulting (ELC), and MiSalud, among others. At the core, however, any certification program must sufficiently teach and assess the national Community Health Worker Core Consensus (C3) competencies and standards established as foundational to the CHW workforce (https://www.c3project.org). There is general consensus among states that regulated CHW certifications need to be required, but also that there needs to be a degree of flexibility to develop unique certification training modes because the context in which CHWs work can differ greatly depending on many regional contextual dynamics. Indeed, the consistent focus for CHW certification program regulation and development is always the expansion of a high quality CHW workforce, while simultaneously acknowledging the need to allow flexibility to account for often very different political, structural, and cultural complexities and constraints.

### Wayne State University Community Health Worker Academy (CHWA)

The following sections describe some of the circumstances and rationale that led to the CHWA's formation, enhancements, and escalation in scope and breadth to prepare and strengthen the CHW workforce throughout an entire metropolitan region. The following overview may help to inform current and future CHW workforce development initiatives that may also see value in capitalizing on the incredible potential of the CHW field to advance population health in their respective communities.

To help address the accelerating CHW workforce demand, we developed the Wayne State University Community Health Worker Academy (CHWA or “the Academy”). During the COVID-19 pandemic, Wayne Health and Wayne State University procured 8 mobile health units (MHUs) to deploy throughout the most impacted areas in and around metropolitan Detroit, first to provide frontline COVID-19 testing, and later to disseminate vaccines (24). As the severity of the pandemic began to decrease, this effort expanded to focus on a model of portable population health (25), leveraged the utility and impact of the MHUs to expand their focus on cardiometabolic risk reduction emphasizing hypertension prevention, detection, and treatment in the communities at highest risk (26, 27). The focus on cardiometabolic risk occurred because of the disproportionate rates of heart disease and stroke in the region, which significantly contribute to premature morbidity and mortality of many community residents. A key feature of this effort was screening and intervention for social determinants of health (SDOH), delivered by CHWs who provided on-site assistance with identified needs.

From this, a vision for expanded engagement was developed with CHWs providing support not just for SDOH but also for selection of activities that patients may be willing to do to better control blood pressure and other risk factors. Termed the PAL2 (Personalized Pragmatic Adaptable Approaches to Lifestyle and Life Circumstance) intervention, this vision was implemented through two multicenter grant programs—Linkage, Empowerment, and Access to Prevent Hypertension (LEAP-HTN—Grant # 878605; Levy, PI), which is part of the American Heart Association RESTORE Health Equity Research Network, and Addressing Cardiometabolic Health Inequities by Early PreVEntion in the GREAT LakEs Region (ACHIEVE GREATER Grant #P50 MD017351: Levy, PI), which is part of the National Institute of Minority Health and Health Disparities Health Equity Action Network). However, those efforts were impacted by the fact that the region had a substantial shortage of CHWs and no facile mechanism to train existing CHWs with the advanced knowledge and upskilling needed to deliver the PAL2 intervention. This critical gap led to a partnership between the university's School of Medicine and the Division of Kinesiology, Health and Sport Studies in the College of Education, specifically the division's Community Health program to provide the education needed to achieve grant goals and shortly thereafter the Community Health Worker Academy launched.

At present, the MHUs are staffed in part by CHWs who are deployed into diverse community settings to implement this “portable population health” initiative, with a focus on hypertension control as well as delivery of other services offered through the units (e.g., cholesterol and diabetes screenings, smoking cessation, COVID testing, influenza and COVID vaccines, etc.). Some of the tasks CHWs executed while “in the field” included, engaging with community members to encourage them to utilize the clinical services being offered, participate in SDOH screenings, access health education resources, or receive referrals to local and regional social services with the potential address SDOH challenges.

The Academy's primary objective at that stage was to create a highly effective and skilled workforce development model that could staff the mobile health units and catapult hypertension reduction across the region. However, in doing so, the Academy was also designed to reduce the attrition rates that commonly plagues the CHW workforce. For instance, in 2021, it was estimated that 12% of the CHW workforce resigned or transferred to alternative employment compared to a 9.3% average attrition rate for all other U.S. occupations (28). To get started, the CHWA mobilized to build personnel capacity and expertise, secure funding for expansion, select a quality and effective certification and continuation education platform, form a diverse cross-sectional Advisory Board, establish aspirational effectiveness metrics, and integrate continuous quality improvement methodologies.

### Funding the academy’s expansion

Serendipitously, as the large-scale hypertension disparities initiative launched and the need to broaden the CHW workforce grew even more dire, the Health Resources & Services Administration (HRSA), a branch of the U.S. Department of Labor, launched the first-ever Community Health Worker Training Program in 2022. The CHWA was fortunate to receive one of 82 national awards in the amount of $2.7 million, which dramatically increased the Academy's capacity to expand and strengthen into a nationally recognized CHW workforce development program.

## CHWA key program components

To expand the Academy, its leadership started by identifying an optimal CHW certification program and recruiting individuals who were interested in gaining employment as a CHW and partnering with agencies that were seeking to begin hiring CHWs or wanted to expand their existing CHW workforce. The Academy recruited a broad, inter-disciplinary staff with many partners and collaborators, including a diverse Advisory Board to provide guidance and oversight.

### The academy’s comprehensive CHW training program

The Academy's leadership, partners, and collaborators collectively agreed that offering a more comprehensive CHW training program was a better path forward than offering only an initial CHW certification program, which is common in many locations. As a result, the CHWA created a three-component program that included (1) an initial CHW certification program that would be authorized by the state of Michigan to credential CHWs, (2) a Healthcare and Public Health Education Catalog to provide additional virtual on-demand training, professional development, and continuing education opportunities for both new and current CHWs, and (3) experiential field placements in the form of either internships or apprenticeships. When CHW trainees successfully complete all three components of the program they receive an Academy Certificate of Completion to complement their official Michigan CHW certification.

The CHWA established affiliate training organization agreements with both MiCHWA and ELC, enabling the Academy to utilize either organization's certification program to certify new CHWs. Both organizations’ CHW certification programs align with the national Community Health Worker Core Consensus (C3) competencies and standards (29) and are authorized to certify new CHWs by the state of Michigan. The Academy employs one master CHW trainer with extensive experience conducting certification programs with cohorts of new trainees and establishes subcontracts with authorized external contract trainers when the volume of training requests exceeds the Academy's capacity. The Academy was designed to be nimble and accommodate the needs and schedules of both CHW employers and new CHW trainees and, as a result, it offers flexible training schedules.

The Academy's leadership, along with its various stakeholders realized from the outset that offering only initial CHW certification programs alone would not adequately prepare CHWs to conduct the highly unique and comprehensive services that are the hallmark of the field, so we developed our Healthcare and Public Health Education Catalog (https://chwacademyeducation.catalog.instructure.com/), which is a virtual online platform offering over 1,200 individual professional development and continuing education courses, as well as 50 Specialized Certificates, in a wide array of healthcare and public health topics (e.g., behavioral health, cancer prevention, cardiovascular disease, diabetes education, cultural competence, motivational interviewing, etc.). CHWs and related healthcare and public health professionals who complete individual courses or Specialized Certificates receive official Wayne State University Community Health Worker Academy Certificates of Completion, which provide users with evidence of their expanded knowledge and skills as industry professionals, as well as advanced credentials to facilitate career mobility.

New CHWs who are completing the Academy's comprehensive program are required to complete a minimum of 24 hours of additional professional education through the Education Catalog. While the initial CHW certification training program provides future CHWs with a global and entry level credential to work in the field, the Healthcare and Public Health Education Catalog is a vehicle for new and already-certified CHWs to obtain customized, in-depth, and user-friendly opportunities to add substantial depth and breadth to their professional skill base and enhance their abilities to advance through the CHW workforce toward expanded career opportunities. The Catalog has emerged as a highly sought after professional development and continuing education mechanism for external agencies to substantially enhance and strengthen not only their CHW workforce, but also their workforce professionals in related healthcare and public health disciplines (e.g., Direct Care Workers, Nursing Assistants, Patient Service Advocates, etc.).

Last, as part of its comprehensive new CHW training program, the CHWA offers two options for experiential field placements, which are designed to provide new CHWs with real-world healthcare and public health work experience supported by the mentorship and supervision of industry professionals. First, new CHWs can elect to participate in 400-hour internships with host organizations that are either paid or unpaid depending on the organization's financial capacities. Second, after attaining their initial CHW certifications, new CHW trainees can also elect to participate in official US Department of Labor (USDOL) Registered Apprenticeships that require one year of paid, on-the-job training hosted by a USDOL-approved CHW registered apprenticeship agency, which also provide extensive mentorship and supervision and a qualified professional mentor.

The Academy offers two experiential field placement options to address the unique training and development needs of the individuals who seek employment as CHWs. For example, the Academy offers internship field experiences to accommodate CHWs who may already be working part-time or volunteering in various healthcare or public health contexts (e.g., faith-based organizations, United Ways, mobile health units, etc.) and are unable to participate in full-time, one-year USDOL Registered Apprenticeships. Whereas some CHW trainees are seeking initial employment as CHWs, others are already employed by a healthcare or public health agency but are required by their employers to attain their certification as CHWs and receive advanced and structured professional education and work experiences to be eligible for reclassification within the agency or attain upward mobility.

Together, the initial CHW certification training program, the required 24 hours of additional professional learning through the Education Catalog, and the experiential field placements through internships or Registered Apprenticeships, provide an in-depth, comprehensive, and tailored system of education and training designed to synergistically result in competent, effective, and high-quality CHWs, and, in turn, a rapidly expanding and greatly enhanced workforce capable of meeting the unique and complex realities of CHW work in a range of healthcare and public health settings. Once again, the foundation of the Academy's three-component CHW training program is to ensure that graduates are fully prepared to implement the national C3 CHW competencies and standards through their work irrespective of the communities and settings in which they provide CHW services.

### Target audience

In promoting the Academy's services and opportunities, aside from recruiting individuals seeking new employment as official CHWs, the Academy leadership has also emphasized an “employer approach” by partnering with diverse health care and public health agencies that employ informal support staff (e.g., patient health advocates, community navigators, etc.), which in most cases execute services that are not eligible for insurance reimbursement. Specifically, employers identify cohorts of existing employees that perform CHW-related services with community residents but because of their informal job classifications are not eligible for insurance reimbursement, and we offer those agencies the opportunity to certify their employees as CHWs, enhance their education and skills in delivering healthcare and public health services, thereby facilitating a higher-performing workforce that is also eligible for employers to seek insurance reimbursement in ways that were unavailable previously. As a result, the Academy's CHW training cohorts (typically 15–20 people each) are comprised of an intentional blending of both individuals seeking first-time employment as CHWs and existing healthcare and public health employees working in agencies that wish to have them earn sanctioned professional credentials, expand and improve their professional education and performance, and achieve status as insurance reimbursement eligible employees.

### The academy’s organizational structure

The CHW Academy is comprised of a multi-disciplinary team of professionals with experience and expertise across the broad spectrum of healthcare, managed care, public health, mental and behavioral health. It directly employs a Director, Associate Director, Operations Manager, Master CHW Educator, and extensive implementation specialists and graduate student support staff. We also have a number of collaborators who are disciplinary specialists (e.g., physicians, nurses, social workers, counselors, etc.). The Director, Associate Director, and most disciplinary specialists are tenured university faculty. The Operations Manager has earned undergraduate and MPH degrees in public health and is currently a PhD candidate. The Master CHW Educator is a former CHW with extensive experience and has served as a CHW educator for many years at various local health departments. The remainder of the Academy team is comprised of undergraduate and graduate student support staff with education and experience working in healthcare and public health settings and in the process of acquiring the advanced knowledge, skills, and experience to become future workforce leaders.

In addition to its core staff, the Academy has also established long-standing collaborations with a variety of industry-related partner organizations in order leverage, but not duplicate, CHW education and workforce services. For instance, the CHWA partners with MiCHWA and ELC, both of which are state-authorized CHW credentialing agencies. As a CHW affiliate training organization with both organizations, the Academy is sanctioned to utilize their state-approved CHW certification programs, and also sub-contract for additional CHW training support as needed based on the volume and demand of individuals seeking employment as credentialed CHWs and the needs of agencies seeking to employ officially certified CHWs. Collectively, between the core CHWA staff, combined with the collaboration and support from key industry partners, the Academy has been able to develop unique and comprehensives educational and advanced credentialling opportunities both for new and existing CHWs.

### CHWA advisory board

Early on, the CHWA recognized the need for professional industry guidance as it continued to develop and expand to ensure its programs would be as relevant and impactful as possible in advancing the CHW workforce. As a result, the Academy recruited a diverse network of CHWs, CHW educators, CHW employers, disciplinary specialists, and other related professionals to form its Advisory Board. The Advisory Board meets quarterly and receives comprehensive updates regarding the Academy's services, the evolution of new educational and credentialing opportunities, and its performance metrics. The board then shares insights about those efforts, including many of the challenges and opportunities related to the expansion and enhancement of CHW workforce. The Academy has found that its Advisory Board provides indispensable guidance and support for its continued operations, evolution, and ultimately success in achieving its mission to support and enhance the CHW workforce in ways that benefit the entire healthcare and public health agenda.

### Preliminary outcome metrics for the academy

Vigilant measurement, tracking, and analysis of the Academy's outcome metrics is one of the key foundations of its success. One method involves continually monitoring the performance and success of its trainees and graduates, especially those supported by its HRSA grant award. For example, the Academy continually monitors and assesses the recruitment, participation, and success of all new trainees as they matriculate through its foundational program, including the impact of the financial supports the grant affords the Academy to offer new trainees (e.g., subsidized CHW certification fees, Education Catalog subscriptions, financial stipends, etc.), as well as other quantitative and qualitative metrics regarding trainee performance in all three program components, trainee attrition trends, trainees’ perspectives of their program participation, and the perceptions of field placement mentor supervisors (i.e., mid- and end-of-field placement surveys), and the perspectives of CHW employers. The Academy employes multiple forms of mixed-method assessments to gain a thorough understanding of the experiences and success of its trainees, as well as the healthcare and public health organizations that host trainees for experiential field experiences, and, upon program completion, hire them as full or part-time CHW employees.

The Academy's program assessments are guided by the established national C3 competencies and standards for the CHW workforce. Program assessments not only evaluate CHW learning and development related to their acquisition of workforce competencies and standards, but also their perceptions of their field placement mentorship and supervision (e.g., mentorship feedback frequency and substance, communication styles, etc.), which are, in the end, critical components leading to CHW development, future success, and feelings about the viability of their careers as CHWs. Comprehensive assessments also provide opportunities for field placement mentor supervisors to provide helpful guidance about the collective process and success of the CHW Academy three-component program in facilitating tangible and credible expansion and advancement of the CHW workforce.

### Continuous quality improvement

In addition to the advice and guidance offered by the Academy's Advisory Board, to ensure that the Academy programs are both sensible and aligned with the needs of the industry, as well as implement and reinforce the established best practices in CHW workforce development, the Academy employes several mechanisms for continuous operational oversight and improvement. In particular, the Academy executes a Rapid Cycle Quality Improvement (RCQI) plan by conducting full-staff bi-weekly meetings to discuss the status and performance of all trainees and cohorts, as well as feedback offered by CHWs, the Master CHW educators, field placement mentor supervisors, CHW employers, and the Advisory Board regarding the extent to which the Academy's programs are operating effectively, yielding positive results and experiences for all stakeholders, and achieving its intended goals and objectives. This involves implementing a PDSA cycle (Plan, Do, Study, Act) to analyze and modify the Academy programs to facilitate optimal performance and success.

## Preliminary findings and discussion

### CHW academy workforce development impact

One source of evidence that demonstrates the impact of the Academy's comprehensive CHW workforce development programs is its success in exceeding its original HRSA grant metrics regarding CHW workforce development, especially in the areas of CHW expansion and enhancement deliverables. In the Academy's initial application for HRSA funding, we indicated that we would increase the CHW workforce by certifying 105 new CHWs through its three-component training program and enhance the knowledge and skills of an additional 35 practicing CHWs, what HRSA refers to as “upskilling.” However, by mid-way through the three-year grant award, the Academy has already certified and graduated 260 new CHWs (with 105 currently in-progress toward program completion), and “upskilled” another 65 already-certified CHWs with many more completing their upskilling programs. The CHWA is recognized as an extremely high performing HRSA-funded program. We anticipate certifying over 400 trainees upon completion of the grant and then continuing our operations via other grant-funded or self-pay mechanisms.

### CHW academy preliminary quality impact

Clearly, demand for the Academy's three-component training program has been high. However, metrics regarding the sheer numbers of program completers is only one way to measure the impact and success of a CHW workforce development program. Another method of assessing program impact and success involves the quality of the Academy's program according to the perspectives of the CHW trainees and their mentor supervisors relative to enhanced CHW learning and performance. One indicator that is assessed throughout the Academy program is the degree to which CHWs perceive their development into quality professionals. Although many CHW trainees are continuing to complete the Academy's three-component program, CHWs who have thus far completed the program, on average, rate their learning and development through mid- and end of field placement assessments between 3.47 and 3.60 (4-point scale) related to the field's established national C3 CHW competencies and standards. These preliminary findings suggest that our CHW trainees believe that they have developed strong knowledge, skills, and confidence to provide services that lead to disease prevention, engage clients in meaningful ways and deliver effective and impactful services, support clients and healthcare providers in “translating” culture-specific services, and offer resources that facilitate health behavior change.

Preliminary qualitative evaluation data also illustrates that CHW trainees believe the Academy's program is of great benefit. The following sample quotes from CHW trainees provides compelling evidence in support of the program's impact from their viewpoints.

“The CHW apprenticeship program opened my eyes to the importance of prevention in this field. To (ensure) that conversations should be had with your clients on needs other than just health care needs.”

“[the program] gave me the confidence to help people.”

“The program taught me how to work more effectively in my community. It gave me a deeper understanding of how healthcare benefits certain communities more than others.”

“I had a lot of hands-on training and it gave me confidence in being an advocate for others.”

Moreover, the Academy's CHW trainees also reported that their mentoring and supervision is typically helpful and supportive. That said, they did occasionally provide feedback indicating some inconsistencies in how mentoring and supervision is executed, and that sometimes additional support could be provided, including technology assistance, which the Academy is beginning to take measures to resolve. This is likely attributed to the wide diversity among the scope of support services at the various agencies where CHW trainees conduct field placement offer, as well as their missions, staffing, and overall operations.

Similarly, CHW trainees’ mentor supervisors generally indicate strong satisfaction with the Academy program. Supervisors provided similar feedback in that for most established national C3 competencies and standards, the average ratings of CHW performance were greater than 3.0 on a 4.0 scale indicating that mentor supervisors believe the Academy program facilitates the preparation of effective CHWs and have a high confidence in their trainees’ future success. Supervisors indicated that CHW time management and specific teaching techniques may be potential areas for training program improvement. An analysis of supervisors’ qualitative data indicated that there was generally a positive sentiment regarding the mentor supervisors’ roles, with supervisors generally feeling that their supervision had been both positive and effective. Strengths highlighted by the mentor supervisors included CHW compassion, resourcefulness, community connections, and leadership qualities. Some of the areas where they believe that CHW trainees could improve included their digital literacy skills, active listening, and cultural exposure.

Another metric that used to determine preliminary program impact involved feedback from the Advisory Board, which showed strong overall satisfaction with the training provided by the CHWA. Feedback in the areas of initial certification (e.g., content included, applicability to certain subgroups), upskilling and specialized trainings (e.g., advanced training courses and Specialized Certificates in the Education Catalog), and field placements (e.g., how best to support mentor supervisors, how much time to allocate to supervision and the best mentorship methodologies to implement, and facilitating CHW accountability) will be utilized to further improve the Academy's training program and facilitate improved workforce development for CHW professionals. Nevertheless, most of the Advisory Board feedback has been positive (e.g., “Our CHWs learned so much and are already applying new knowledge and skills with clients,” “The Academy is giving people such great opportunities to learn what they need to be successful as CHWs and in public health”).

A related metric being used to understand the impact of the Academy's programs is the interest in partnerships the Academy has receive by other units and program across the university, as well as by external agencies. Partnerships have varied from the inclusion of the Academy's programs in basic science grant proposals and other workforce development initiatives (e.g., aging adult formal and informal caregiving). Some organizations want to purchase subscriptions to the Academy's Healthcare and Public Health Education Catalog, while others want to partner with the CHWA on various healthcare and public health projects that seek to integrate CHWs into care delivery models and study their impact on addressing various chronic health conditions (e.g., hypertension, sleep-disordered breathing, mental health, etc.) or other unique populations and settings (e.g., geriatric care, senior living care, youth mental health). There is clearly a high demand throughout the university and community for collaborations to utilize qualified and effective CHWs in healthcare and public health initiatives seeking to advance health equity and expand and strengthen the overall healthcare and public health workforce.

### Implications and applications

Across all facets of the Academy's preliminary assessment of its impact, the common theme was positive perceptions of the quality and impact of its comprehensive training model. Feedback from all constituents reflects high satisfaction with the CHWA program. This is remarkable given the Academy's leadership was uncertain as to how the existing workforce and industry would respond to such a comprehensive training model, especially given that many existing training models require little if any advanced education and/or field placements, beyond conventional initial CHW certification programs that are far more general in nature. The addition of CHW and mentor supervisor evaluations and other performance monitoring metrics has been particularly beneficial in assessing the Academy's programs and effectiveness, which are also not common in most CHW training programs.

It is encouraging, however, that the Academy is also experiencing strong demand from both healthcare and public health researchers conducting population health interventions with CHWs as core components of intervention implementation. These opportunities present the Academy with a range of viable and expanded funding opportunities. Interest from external organizations in partnerships with the Academy was not unexpected; however, the level of interest and number of multi-faceted agencies has been remarkable and further illustrates the viability and long-term possibilities for the Academy to place key roles in providing unique services to advance population health through innovative mechanisms.

One refinement that the Academy is implementing is creating CHW mentor supervisor training program best practices guidance and attempting to standardize the mentoring and supervision that CHW receive whether during training field placements or during employment. While a few CHW mentor supervisor training programs currently exist, an emphasis on standardizing mentoring practices is important to the future growth of the field, particularly to encourage retention. Other performance metrics that the Academy is currently analyzing include the optimal content and duration of CHW initial certification training programs, the most effective types of and duration of field placements, and the most effective peer mentorship approaches. The Academy is also tracking the employment of its graduates to be able to understand and identify additional methods to facilitate the long-term employment retention and success of new CHWs.

Some of the nuances and potential limitations of this work may include that many CHW trainees who participate in the Academy's programs do so at their employers’ request because they are highly motivated to have their employees officially certified, in particular to obtain Medicaid reimbursement for their CHW services. That said, however, all CHWs that participated in the Academy's training program did so of their own volition and were offered the opportunity as part of their employment and continuing professional development. Individuals who voluntarily joined the Academy's CHW program did so because of their own personal motivation to obtain the skills and credentials to obtain viable employment in the healthcare and public health workforce. It could also be seen as a limitation that the Academy primarily serves one U.S. state and especially a large metropolitan region in and around one of the most underserved cities in the state that is plagued with a host of health disparities. Nevertheless, while there are many variations of CHW training programs, there is a high degree of shared perspectives regarding the role and function of CHWs, and having this degree of consensus across the field is a strength and foreshadows optimism for its future viability.

Additional evolution of our CHW Academy will continue to be informed by not only workforce development trends but lessons learned through continuous process and outcome assessments and subsequent program improvements. The long-term goal of the CHWA is to be as responsive as possible to the ever-expanding and evolving needs for a highly qualified CHW workforce to improve individual, community, public, and population health and reduce the health inequities that occur particularly in underserved communities that have a long history of marginalization and negative, yet preventable, health outcomes.

In summary, the extensive participation from individuals and organizations in the Academy's CHW services and the positive data-based assessments documenting its success indicates that the Academy has been well-received and is having a positive impact on the communities in most need of the unique services that CHWs provide across the healthcare and public health spectrum. While contexts vary between states and countries that may have different healthcare and public health systems, policies, and workforce structures, the Academy's training and development model has strong potential to be replicated in many similar and diverse contexts. It may be of interest to anyone wishing to embrace the CHW profession in their aspirations to facilitate population health and health equity.

## Data Availability

The data used in this article is available upon reasonable request from the corresponding author.
